# Cost evaluation of a nurse coordinated outpatient parenteral antimicrobial therapy (OPAT) program

**DOI:** 10.1017/ash.2023.526

**Published:** 2024-01-03

**Authors:** Huiwen Deng, Alan E. Gross, Andrew B. Trotter, Daniel R. Touchette

**Affiliations:** 1 Department of Pharmacy Systems, Outcomes and Policy, University of Illinois Chicago College of Pharmacy, Chicago, IL, USA; 2 Department of Pharmacy Practice, University of Illinois Chicago College of Pharmacy, Chicago, IL, USA; 3 Division of Infectious Disease, Department of Medicine, University of Illinois Chicago College of Medicine, Chicago, IL, USA

## Abstract

A structured, nurse-driven outpatient parenteral antimicrobial therapy (OPAT) program within an academic healthcare system was associated with reduced odds of 60-day unplanned OPAT readmissions and costs after hospital discharge. These findings may facilitate justifying additional resources for OPAT programs to improve care while decreasing costs.

## Introduction

Compared with inpatient parenteral antimicrobial therapies, outpatient parenteral antimicrobial therapy (OPAT) provides a more cost-effective option for delivering intravenous antimicrobials to medically stable patients.^1–3^ However, OPAT carries risks like catheter complications and requires a multidisciplinary team for safe and effective treatment management.^1,3^

Before 2017, the University of Illinois Hospital and Health Sciences System (UI Health) OPAT program was overseen exclusively by infectious disease physicians without administrative staff for care coordination.^4^ The integration of an OPAT nurse in October 2017, who oversees treatment coordination, monitoring, and documentation, led to a 10% reduction in unplanned OPAT-related readmissions during the OPAT therapy, as identified by Agnihotri et al.^5^ However, this study did not explore readmissions at standardized follow-up periods post-discharge or the accompanying costs of these readmissions between the pre- and post-intervention programs. The primary objectives of this report were to quantify odds ratios (ORs) of unplanned OPAT-related readmissions within 30 and 60 days post-discharge between the pre- and post-intervention programs and to evaluate associated costs from the payers’ perspective.

## Methods

### Setting, participants, and intervention

This was a retrospective observational cohort study of patients who received OPAT after hospital discharge at UI Health. The study design and participant selection were described in previous studies.^4,5^ In brief, we included patients aged 18 years and older, who received OPAT through a peripherally inserted central catheter for at least two days and had an infectious diseases consultation during the index hospitalization. Patients with cystic fibrosis were excluded. We collected UI Health records from January 2012 to August 2013 for the preintervention program and from October 2017 to January 2019 for the post-intervention program. The Institutional Review Board of the University of Illinois at Chicago approved this study.

#### Outcomes

This study’s primary outcomes were ORs for unplanned OPAT-related hospital readmissions within 30 and 60 days after hospital discharge, along with the associated costs. Two infectious disease clinicians reviewed OPAT patients’ medical records to indentify such readmissions, which were OPAT-related if they resulted from complications like infection recurrence, adverse drug reactions, or catheter-related problems. Hospital reimbursement costs for readmissions within 60 days were calculated and adjusted to 2019 US dollars, with cumulative costs for patients experiencing multiple OPAT-related readmissions.

### Statistical analysis

We compared 12-month baseline characteristics linked to OPAT-related readmissions and associated costs before and after the nurse-integrated structured OPAT program was established. Multivariate logistic regression was employed to calculate readmission ORs with 95% CIs, and Hosmer–Lemeshow test to evaluate the goodness of model fits. The model covariates were chosen based on a change-in-estimate criterion with a cutoff of 10%.^6^ To analyze cost associations with unplanned readmissions, we utilized a zero-inflated two-part model for its semicontinuous distribution. We incorporated logistic regression for the high zero cost prevalence and a gamma-distributed generalized linear model with log link to count for nonzero cost data distribution variability.^7^ Akaike’s Information Criteria and Bayesian Information Criteria were used to select the covariates in the two-part model, with lower values indicating better goodness of model fits. Margins estimation was then applied to predict the adjusted average readmission costs in both programs using model prediction equations.^8^ A *P* value of less than 0.05 was indicative of statistically significant results. All analyses were conducted using Stata version 17 (College Station, TX: StataCorp LLC).

## Results

The study included 428 eligible patients in total, with 73 from the preintervention group and 355 from the post-intervention group. Patient demographics are listed in Table 1.

**Table 1.** Descriptive characteristics of patients enrolled in the preintervention and post-intervention OPAT programs

	Preintervention OPAT program (*N* = 73)		Post-intervention OPAT program (*N* = 355)		Standardized difference
**Age, years**, median (IQR)	52	47–67	57	43.5–60.5	−0.30
**Insurance**, *n* (%)					
Government-funded insurance	47	64.4	259	73	−0.26
Private insurance	23	31.5	79	22.3	
No insurance	3	4.1	17	4.8	
**Charlson comorbidity score**, median (IQR)	2	0.5–3.5	3	1.5–4.5	−0.53
**Previous hospitalizations for any cause within past year**, *n* (%)	36	49.3	182	51.3	−0.04
**Any intensive care unit visit during index hospitalization**, *n* (%)	31	42.5	98	27.6	0.31
**Lengths of stays of index hospitalization, days**, median (IQR)	8	4.5–11.5	7	4–10	0.09
**Planned duration of OPAT treatment, days**, median (IQR)*	42	32–52	36	22.5–49.5	0.13
**Outpatient treatment duration**, median (IQR)	30	19–41	25	12–38	0.01
**OPAT indications**, *n* (%)					
Bone and joint infection	41	56.2	133	37.5	−0.49
Central nerve system infection	13	17.8	34	9.6	
Skin soft tissue infection	6	8.2	29	8.2	
Genital/urinary tract infection	2	2.7	36	10.1	
Intra-abdominal infection	2	2.7	34	9.6	
Others	9	12.4	89	25.1	
**Location of administration**, *n* (%)**					
Ambulatory	44	60.3	191	53.8	−0.13
Nonambulatory	29	39.7	164	46.2	
**Use of vancomycin**, *n* (%)	39	53.4	110	31	0.46

*Excluded 1 observation because of numeric error in the record.

**Ambulatory sites include home and infusion center; nonambulatory sites include skilled nursing facility, subacute rehabilitation facility, and unknown sites.

### Unplanned 30- and 60-day OPAT readmissions

After the implementation of the structured OPAT program, the unplanned 30- and 60-day OPAT-related readmission rates decreased from 15.1% and 17.8% to 5.9% and 6.2%, respectively (Table 2). The use of vancomycin during 12-month baseline period was identified as an independent predictor of readmission based on the prespecified change-in-estimate criterion. Upon adjusting for vancomycin use, the adjusted estimates suggested a nonsignificant 52% reduction in the odds of 30-day readmissions (Table 3, 95% CI: 0.22–1.05, *P* value: 0.067) and a significant 58% reduction in the odds of 60-day readmissions (95% CI: 0.19–0.91, *P* value: 0.028) for the post-intervention group relative to the preintervention group. The Hosmer–Lemeshow tests validated the good fit of the models, indicated by nonsignificant *P* values exceeding 0.05.

**Table 2.** Frequencies of 30-day and 60-day unplanned OPAT-related hospital readmissions between OPAT programs, *n* (%)

	Preintervention OPAT program (*N* = 73)	Post-intervention OPAT program (*N* = 355)	Standardized difference
30-day hospital readmission	11 (15.1%)	21 (5.9%)	0.28
60-day hospital readmission	13 (17.8%)	22 (6.2%)	0.33

**Table 3.** Association between OPAT programs and 30-day and 60-day unplanned OPAT-related hospital readmissions

	Odds ratio (post- vs preintervention program)	95% CI		*P* value
30-day hospital readmission*	0.48	0.22	1.05	0.067
60-day hospital readmission*	0.42	0.19	0.91	0.028

*Adjusted for use of vancomycin.

### Associated readmission costs

Table 4 delineates the relationship between OPAT program types and the costs of unplanned readmissions within 60 days post-discharge. Compared to the preintervention group, post-intervention patients exhibited a lower likelihood of incurring any OPAT-related hospital cost (OR = 0.37; 95% CI: 0.17–0.82; *P* value: 0.015). However, for patients readmitted due to OPAT, the costs of hospitalization did not significantly differ between the programs (OR= 0.93; 95% CI: 0.49–1.74; *P* value: 0.810). After adjustments for age and any intensive care unit visit during the initial hospital stay, the average predicted readmission costs were $5,685 for the preintervention group versus $2,201 for the post-intervention group, reflecting a 61.3% cost reduction.

**Table 4.** Adjusted ratios for unplanned OPAT-related readmission cost between pre- and post-intervention programs*

	Odds ratio (post- vs preintervention program)	95% CI		*P* value
Any readmission costs**	0.37	0.17	0.82	0.015

	Cost ratio (post- vs preintervention program)	95% CI		*P* value
Amount of readmission costs, conditional on any spending**	0.93	0.49	1.74	0.810

**Adjusted for age and any intensive care unit visit during index hospitalization.

## Discussion

Our findings corroborate those of Agnihotri et al., which evaluated unplanned OPAT-related readmissions at any time.^5^ At 60 days, unplanned OPAT-related readmissions were lower in the post-intervention program with a similar proportion of patients hospitalized to what was previously reported. However, the ORs of readmission between the two programs were not statistically different at 30-days, potentially due to insufficient power to detect the smaller observed difference in hospitalizations between the two programs. The costs of OPAT-related readmissions, but not readmissions due to OPAT, were also substantially lower. To the best of our knowledge, this is the first study to examine the comparative costs for two OPAT programs with different care coordination structures.

Previous research on the cost-effectiveness of nurse-facilitated disease management programs has produced mixed results.^9,10^ A systematic review of transitional care from hospital to home for diverse patient groups, such as those recovering from cancer surgery and heart failure patients, showed that nurse-led coordination significantly lowers readmission rates and associated costs when contrasted with noncoordinated care approaches.^9^ Conversely, an evaluation of 34 Medicare programs catering to patients with chronic conditions indicated no reduction in hospital readmission rates after integrating nurse coordination, except in cases where nurse coordinators had considerable direct interactions with both physicians and patients.^10^ Costs in these nurse-coordinated programs were generally unchanged or higher after accounting for labor and program fees.^10^ It’s important to note that the costs evaluated in prior studies were more comprehensive, whereas our study specifically examines OPAT readmission-related costs.

The study is subject to several limitations. Its retrospective nature and single-center scope may affect the generalizability of the findings. We also compared past and recent OPAT programs, risking confounding due to historical changes in healthcare practices. Although demographic predictors of outcomes were accounted for, the potential for residual confounding remains, and systematic variations may persist among patients enrolled in different programs. The analysis captured only follow-up care provided at UI Health. Additionally, the cost analysis focused exclusively on unplanned OPAT readmission reimbursements, excluding other related expenses and program fees, and was conducted solely from the payer’s perspective. Future research should expand to evaluate the broader economic impact of nurse-coordinated OPAT programs, incorporating indirect costs and from patient, hospital, and societal perspectives.

## Financial statement

UIC Center for Clinical and Translational Science UL1TR002003

## Related article

This work is an extension of a previously published article, entitled “Decreased hospital readmissions after programmatic strengthening of an outpatient parenteral antimicrobial therapy (OPAT) program,” which was published on February 21, 2023, with a DOI https://doi.org/10.1017/ash.2022.330.
